# The Western Ontario Shoulder Instability Index (WOSI): validity, reliability, and responsiveness retested with a Swedish translation

**DOI:** 10.3109/17453670902930057

**Published:** 2009-04-01

**Authors:** Björn Salomonsson, Susanne Ahlström, Nils Dalén, Ulf Lillkrona

**Affiliations:** Division of Orthopedics, Karolinska Institutet Danderyd HospitalStockholmSweden

## Abstract

**Background and purpose** The WOSI score questionnaire is a tool designed for self-assessment of shoulder function for patients with instability problems. We made a translation into Swedish and retested the score by analyzing the psychometric properties validity, reliability, and responsiveness.

**Patients and methods** 3 patient materials were used for the assessment: (A) a follow-up on a group of 32 patients more than 8 years after having primary posttraumatic shoulder dislocation. Evaluation of Pearson’s correlation coefficient between WOSI and Rowe score and for test-retest reliability was made; (B) 22 patients, treated with a surgical stabilization of the shoulder at our department, were evaluated with Pearson’s correlation coefficient between WOSI and EQ-5D, and between WOSI and a VAS-scale of general shoulder function. Also, Cronbach’s alpha, effect size, and floor, and ceiling effects were analyzed; (C) 45 students with healthy shoulders (reference group) had their WOSI score determined.

**Results** The construct validity (Pearson’s correlation coefficient) was adequate (0.59) between the WOSI score and the Rowe score. The agreement with an ICC value (test-retest) for the WOSI score was excellent (0.94). Cronbach’s alpha (internal consistency) was satisfactory, with 0.89 preoperatively and 0.95 postoperatively. All 22 patients in group B reported improvement in the WOSI score (mean 29%). Responsiveness was excellent, with an effect size of 1.67 for the WOSI score. There were no floor or ceiling effects for the Swedish WOSI score. The mean WOSI score from group C with 45 normal healthy shoulders was 96%, with no floor but high ceiling effects.

**Interpretation** WOSI score does not require an examination of the patient and can be administered by mail. The high ICC and sensitivity makes it able to monitor an individual patient’s progress. At this retest, the WOSI score has good validity, a high degree of reliability, and a high degree of responsiveness, all at the same level as in the original publication. We recommend the WOSI when evaluating patients with instability problems.

Several instruments have been developed to determine the outcome of orthopedic management of shoulder conditions, including instability problems, but most are derived from clinical data and depend on the judgments of an examiner. Instability of the shoulder leads to special problems of assessment, as the symptoms are often intermittent and are characterized less by pain than by the anticipation of problems arising in association with certain activities. Instruments are required that concentrate on the patient’s subjective viewpoint about the outcome. Such a patient-evaluated disease-specific quality of life scoring system has been developed for shoulder instability patients by Kirkley et al. (1998), who evaluated the properties of the score with the original English version, the Western Ontario Shoulder Instability Index (WOSI). We have translated the WOSI score questionnaire into Swedish according to the descriptions by Guillemin et al. (1993). It is advisable to establish the psychometric properties of any translated score, at least until there are numerous translated and evaluated versions in different languages.

It is essential to use outcome instruments with psychometric properties that have been retested. The important psychometric properties include validity, reliability, and responsiveness. Validity states whether an instrument actually measures what it intends to measure, and reliability refers to the reproducibility of the outcome measure. Responsiveness is the ability of the score to monitor changes (sensitivity of the score to measure change over time). The ideal scoring system should also be simple, effective, and easy to use so that all orthopedic surgeons can incorporate the tool into their practice.

The purpose of this study was to retest the psychometric properties of the WOSI score using a Swedish translation and to compare the WOSI score with the Rowe score.

## Patients and methods

We used 3 different patient materials for assessment of the score.

## Group A (n = 32)

During the period 1994–1997, 45 patients with a primary traumatic shoulder dislocation were treated by closed reduction and had radiographs taken immediately after reduction. More than 8 years later, all patients were contacted by mail and asked to complete a questionnaire and a self-evaluating shoulder instability quality of life score (WOSI), and they were also asked to participate in a clinical examination of their shoulder including a Rowe score. Of the 45 patients, 32 patients returned the WOSI score on 2 occasions within 2 months, and also underwent a clinical examination with a Rowe score (Rowe 1988). This group of 32 patients was used for evaluation of the criterion validity, expressed as Pearson’s correlation coefficient between WOSI and Rowe score, and test-retest reliability. All 32 patients had had at least one episode of instability; 23 were had not been operated and 9 had been operated before the follow-up. There were 7 women and 25 men, and their mean age had been 36 (17–69) years at the time of the primary dislocation.

### Group B (n = 22)

For other statistical evaluations, we used a second group of 22 patients. They had all been treated with a surgical stabilization of the shoulder at our department. They filled in the WOSI score and EQ-5D (a global health measure consisting of 5 items) (Brooks 1996) preoperatively and by mail 6 months postoperatively. Postoperatively, the patients were also asked to grade their satisfaction in categories, as well as general shoulder function and their own perception of the effect of the treatment on VAS scales. There were 9 women and 13 men, and their mean age was 35 (20–60) years. This group was used for the evaluation of Pearson’s correlation coefficient between WOSI and EQ-5D, as well as a VAS-scale of shoulder function, and also for evaluation of internal consistency by Cronbach’s alpha, effect size, SRM (standardized response mean) and floor and ceiling effects.

### Group C (n = 45)

As a reference of how the normal population would score in the WOSI score, we asked 45 students who reported having a healthy shoulder (without any known instability or other shoulder problems) to fill out the WOSI questionnaire. There were 28 women and 17 men and mean age was 27 (21–42) years.

### The Western Ontario Shoulder Instability Index (WOSI)

The WOSI score instrument (Kirkley et al. 1998) consists of 21 items. The patient is asked to grade the function of a specific item on a horizontal visual analog scale from 0 to 100 mm. The questions are divided into 4 sections (domains). There are 10 questions addressing physical symptoms and pain. Sport, recreation, and work are addressed in 4 questions. There is a domain with 4 questions dealing with lifestyle and social functioning, and another domain for emotional well-being with 3 questions. Each question results in a number between 0 and 100 and the total score may be presented as a number between 0 and 2,100 points (where 0 represents no deficit and 2,100 the worst). The score can also be presented as percentage of a normal healthy shoulder, which may be more clinically useful. We made a translation and cross-culture adaptation of WOSI to Swedish (see Supplementary data) according to the guidelines presented by Guillemin et al. (1993). These guidelines include several steps: (I) translation from the original score to the new language by two independent translators; (II) preparation of a consensus translation; (III) back-translation of the consensus version by a native speaker of the original language, to check for any discrepancies; (IV) preparation of a final version by consensus; (V) testing of the final translation on patients by selected users, looking for practical problems or possible misunderstandings, prior to acceptance of the translation for use.

### Validity

The ability of a score to measure all the intended aspects of the actual condition in such a way that it is applicable to all patients with that condition is called construct validity. Since there is not one single accepted manner to verify validity, it must be done in multiple ways. One important aspect concerning verification of validity is to ensure that the conclusions from the use of the measure in different studies are subsistent over time (Pynsent 2001).

Criterion validity is used express whether the instrument agrees with the truth—or with a “gold standard” if no absolute truth can be measured. This has been verified in the initial process of developing the score (Kirkley et al. 1998), but we decided to complement this with a comparison of a Swedish version of the Rowe score of 1988. This was done in patient group A (n = 32). Pearson’s correlation coefficient was used to correlate the scores with each other.

For group B (n = 22), the EQ-5D and the patient self-assessment of their overall shoulder function on a VAS scale was compared to the WOSI score. Our hypothesis was that the WOSI score would agree better with the subjective VAS scale of shoulder function than with the overall health measurement by the EQ-5D as a measure of construct validity. Measurements were performed on the values of the postoperative test, as well as on the improvement in the different assessments.

Content validity assesses whether the items measure what they claim to measure, and also if they measure the full range of the actual question. The floor and ceiling effects are often used to assess this. These effects are the phenomenon that the results of an item may cluster in the highest or lowest result group. The distribution of the results in the different groups of each item, both pre- and postoperatively, are presented and evaluated. For this study of the Swedish WOSI, the values 0 to 1 were regarded as the lowest possible values, and 99 to 100 were regarded as the highest possible. The floor and ceiling effect is also considered important for the analysis of responsiveness. Floor and ceiling effects are presented in responsiveness as they indicate limits to the range of detectable change. Beyond the limits, no further improvement or deterioration can be observed. This was studied in group B (n = 22) both preoperatively and postoperatively, and also for the reference group C (n = 45).

Convergent validity is assessed by the correlation between items that make up the score, measuring internal consistency validity. Cronbach’s alpha was used to measure internal consistency in group B (n = 22), both preoperatively and postoperatively. Cronbach’s alpha increases as the number of items in the score increases, and comparison of alpha levels between scores with different numbers of items is not useful. When using Cronbach’s alpha on a composite rating scale made up of several items, it is also a measure of reliability.

### Reliability

The test-retest reliability was expressed by measuring the agreement over time by intraclass correlation (ICC) between 2 measurements (without episodes of instability between the measurements) less than 2 months apart, more than 8 years after the initial primary dislocation in group A (n = 32). The assessment was performed for all 21 separate items, as well as for the domains and the total WOSI score.

### Responsiveness

Responsiveness (sensitivity) to change between measurements before and after treatment (over time) was analyzed in 2 ways.

Effect size is a difference between the postoperative mean values and the preoperative mean values divided by the preoperative standard deviation. In this way, the outcomes of related but not identical scores could be analyzed. This was studied for WOSI, EQ-5D, and VAS-function in group B (n = 22). We also measured the SRM (standardized response mean) as a difference between the postoperative mean values and the preoperative mean values divided by the standard deviation of the difference.

### Statistics

The methods were chosen for comparison with the original score (Kirkley et al. 1998), and the methods used were Pearson’s (product-moment) correlation coefficient, the Intraclass Correlation Coefficient (ICC), Cronbach’s alpha, effect size, SRM (standardized response mean), and description of floor and ceiling effects. See Table 1 for the outcome measures criteria (Salter et al. 2005).

**Table 1.** Criteria for outcome measures

	Excellent	Adequate	Poor
Pearson’s correlation (criterion validity)	≥ 0.60	0.31–0.59	≤ 0.30
Floor and ceiling effects (content validity)	0%	≤ 20%	>20%
ICC (test-retest)	≥ 0.75	0.40–0.74	< 0.40
Cronbach’s alpha (internal consistency, convergent validity)	≥ 0.80	0.70–0.79	< 0.70
Effect size (sensitivity to change)	≥ 0.80	0.50–0.79	< 0.50
Standardized response mean (sensitivity to change)	≥ 0.80	0.50–0.79	< 0.50

Based on tables presented in Salter et al. (2005) http://www.ebrsr.com/modules/module21.pdf

The study was approved by the Ethics Committee of Stockholm (2003-557 and 2006/54-31/2) and was conducted in accordance with the Helsinki Declaration.

## Results

### Validity

The criterion validity, expressed as Pearson’s correlation coefficient, was 0.80 between the WOSI score and function assessed by VAS. For the domain physical symptoms it was 0.70, for sport it was 0.68, for lifestyle 0.75, and for emotions it was 0.76, as correlated to function assessed by VAS. The Pearson’s correlation coefficient was 0.59 between the WOSI score and the Rowe score postoperatively (Table 2).

**Table 2.** Statistical evaluation of the validation for domains and the WOSI score. Test-retest reliability for domains and the WOSI score, and correlation to Rowe score

	Pearson’s correlation coefficient				Floor / ceiling effects	
Domains WOSI	VAS **^a^**	EQ-5D **^a^**	ROWE **^b^**	ICC **^b^**	Preop. **^c^**	Postop. **^c^**
Physical	0,70			0.90	No	5% (1) C
Sport	0.68			0.85	5% (1) F	5% (1) C
Lifestyle	0.75			0.89	No	5% (1) C
Emotion	0.76			0.91	No	14% (3) C
WOSI score	0.80	0.44	0.59	0.94	No	No
Effect pre/post	0.59	0.45				
EQ-5D					27% (6) C	59% (13) C

**^a^** Group B, n = 22. The difference in WOSI score correlated to the difference in a single VAS scale (subjective shoulder function and EQ-5D as effect of the treatment). **^b^** Group A, n = 32. Test-retest reliability by intraclass correlation coefficient (ICC), and correlation of the WOSI score to Rowe score.

**^c^** Group B, n = 22. F = floor, C = ceiling (number of answers in F/C in brackets). Floor (worst possible) considered as being 0–1%. Ceiling (best possible) considered as being 99–100%.

There were no floor or ceiling effects for the WOSI score. Preoperatively, the only floor effects were 1/22 for the sport domain and there were no ceiling effects. Postoperatively, there were no floor effects and low but adequate ceiling effects in all domains (Table 2).

Analysis for separate items showed 2 items from the physical symptoms domain that had several answers in the ceiling region already preoperatively. For item 2, asking about pain, there were 9/22 preoperatively and 12/22 postoperatively. For item 7, asking about discomfort in the neck muscles, there were 8/22 with ceiling effects preoperatively and 10/22 postoperatively. Preoperatively, there were 36 item answers (out of 462) with floor effects as compared to 5 postoperatively. Postoperatively, there were 114 of 462 item answers with ceiling effects as compared to 47 answers preoperatively.

### Reliability

**Figure 1:**
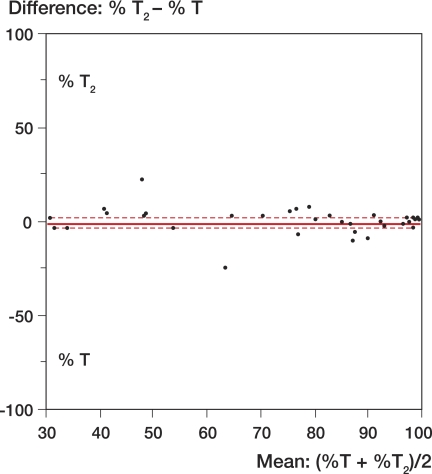
Agreement between the 2 measurements (T and T_2_). Group A (n = 32): test-retest as a difference plot of the total WOSI score (as % of a normal shoulder). The difference compared to mean score for each pair. Correlation was 0.94 between the 2 measurements.

The ICC value (test-retest) for the entire score was 0.94 (Table 2); the agreement is also shown as a plot (Figure 1). For the different domains, see Table 2. The value for the 21 different items varied between 0.75 and 0.97.

Cronbach’s alpha (internal consistency) for WOSI score varied between 0.88 and 0.90 for the 21 different items preoperatively and between 0.94 and 0.95 postoperatively. Among the domains, the preoperative lifestyle domain had the lowest value of Cronbach’s alpha (0.56) (Table 3).

**Table 3.** Internal consistency for domains, the WOSI score, and EQ-5D. Group B, n = 22: internal consistency by Cronbach’s alpha for domains and the WOSI score

Domains	Items	Mean	SD	Range	Cronbach’s alpha
*Preop. (n = 22)*					
Physical	10	60	22	16–91	0.89
Sport	4	44	25	0–96	0.84
Lifestyle	4	52	21	14–89	0.56
Emotion	3	28	21	2–95	0.78
WOSI score	21	51	18	13–87	0.89
EQ-5D	5	0.80	0.18	0.26–1	
*Postop. (n = 22)*					
Physical	10	85	13	57–99	0.90
Sport	4	78	21	23–99	0.91
Lifestyle	4	82	18	31–99	0.79
Emotion	3	63	32	9–100	0.91
WOSI score	21	80	17	45–98	0.95
EQ-5D	5	0.91	0.12	0.73–1	

### Responsiveness

All 22 patients reported improvement in the WOSI score (mean 29%). The effect size for WOSI score was 1.67, and for the domain physical symptoms it was 1.15, for sport 1.15, for lifestyle 1.43, and for the domain emotions it was 1.64. The SRM (standardized response mean) was 1.40 for the WOSI score (Table 4).

**Table 4.** Effect of treatment. Group B, n = 22: responsiveness analyzed by effect size and standardized response mean for WOSI in domains, WOSI score, and EQ-5D

Domains	Effect size	Standardized response mean
Physical	1.15	1.09
Sport	1.15	1.01
Lifestyle	1.43	1.28
Emotion	1.64	1.11
WOSI score	1.67	1.40
EQ-5D	0.65	0.71

### Reference group

Table 5 shows a breakdown of the answers in the WOSI score from the reference group with normal, healthy shoulders (mean total score of 96%; no floor effects and high ceiling effects in all items).

**Table 5.** Reference group, domains, and the WOSI score. Group C, n = 45: the WOSI score for the students without shoulder disorder. Presented as percentage of a healthy shoulder (maximum 100%) for domains and the WOSI score

Domains	Items	Mean (%)	Median (%)	Range	n at ceiling **^a^**	% at ceiling **^a^**
Physical	10	94	96	59–100	12	27%
Sport	4	98	99	86–100	32	71%
Lifestyle	4	98	99	89–100	29	64%
Emotion	3	96	99	52–100	26	58%
WOSI score	21	96	97	73–100	15	33%

**^a^** No floor effects; ceiling considered as being 99–100%.

## Discussion

This first psychometric assessment of a translated WOSI score shows that it is a valid, reliable and sensitive instrument for assessment of groups of patients, and in some respects also for individual patients with shoulder problems associated with instability.

The Swedish WOSI score had acceptable criterion validity as it correlated well with the Rowe score. It is interesting to note that the Pearson’s correlation coefficient with Rowe score, 0.59, is very close to the value of 0.61 presented by Kirkley for the original English version of the score (Kirkley et al. 1998). The correlation with the subjective VAS shoulder function was higher than that with the Rowe score. This could be because a large proportion of the Rowe score is attributed to frank instability and range of motion, which minimize the effect of many other symptoms that can be relevant for patients’ subjective evaluation of their function. As expected, the EQ-5D, which is a global measure of health, was found to have a low correlation to the disease-specific WOSI.

The test-retest reliability of the WOSI was high, with ICC values for the different items of between 0.75 and 0.97. As an ICC value of 0.9 is regarded as acceptable for reliable decision making, even for individual patients (Bot et al. 2004), the WOSI score can be used for that as well as the separate domains—except for the domain sport, recreation and work. The finding that the ICC was 0.94 for the WOSI score compares well with the ICC of 0.95 in the original paper. This indicates that the translation did not dramatically change the properties of the score.

All 22 patients reported improvement in the WOSI score, and this is in agreement with the large effect size of 1.67 for the WOSI score. The SRM (standardized response mean) was 1.40, and this is higher than that of 0.93 in the original presentation by Kirkley et al. (1998).

Improvement in outcome of shoulder stabilization surgery may be difficult to detect when only measuring new instability symptoms, but may anyhow be of clinical relevance. To visualize moderate differences, it is necessary to have instruments that are highly sensitive to clinical change. Our study shows that the translated WOSI has a very high sensitivity, expressed as effect size as well as SRM (standardized response mean). One advantage of a highly responsive score is that in clinical trials fewer subjects are required to show a statistically significant difference between treatment groups (Kirkley et al. 1998). In comparison to other shoulder scores that have been investigated, the WOSI score does well regarding sensitivity to change for instability disorders of the shoulder (Romeo et al. 1996, Kirkley et al. 2003, Kocher et al. 2005). A high responsiveness also indicates that a score is valid, which is supported by the high content validity shown by minimal floor and ceiling effects.

As expected, the mean and median values were very high for the reference group (C) with very high ceiling effects in all items. Still, some questions can be raised about the suboptimal score value in students with no shoulder problems. It must be remembered that several items relate to symptoms that not are entirely shoulder-associated—but that could in any case be relevant and sensitive for a patient with a history of shoulder instability. For example, questions 5, 6, and 7 relate to clicking, stiffness, and symptoms from neck muscles; these are not necessarily related to shoulder disorders or impaired function. The fact that the value of the score is not 100% for all individuals with healthy shoulders supports the idea that the score is also sensitive for patients with modest symptoms.

One limitation of our study was that there was heterogeneity among the patients. Although they had all had instability problems, they consisted of 2 small study groups. A question still remaining to be answered concerns the size of the minimally clinically important change in the WOSI score. The preliminary data from the development of the score indicate that an individual change in the WOSI score of 10% represents a minimally clinically important change, and that a moderate improvement in quality of life would be about 22% (Kirkley et al. 2005).

Traditional physician-based parameters such as motion and strength do not provide direct evaluations of shoulder function, which is essential for outcome assessment. An ideal scoring system should be strongly weighted towards functional outcome; the patient’s point of view must be prioritized (Romeo et al. 1996). In this score evaluation, there are convincing indications that the score also assesses important symptoms other than instability itself.

The WOSI score is user-friendly and could be administered by mail, and the high ICC and sensitivity makes it suitable for monitoring the progress of an individual patient. It is also designed for clinical trials and is valid for comparing and even aggregating cohort studies. Patient-evaluated outcome measures are intended to supplement and not replace conventional measures of outcome such as range of motion, strength, and other cardinal symptoms (Lillkrona 2008).

At the retest, the WOSI score had good validity and high reliability with internal consistency and high responsiveness, all at the same high level as in the original publication. We therefore suggest that the WOSI score should be included when evaluating patients with instability problems.
